# Synergistic antibacterial effect of CATH-2 and D-amino acids against mastitis causing gram-positive bacteria

**DOI:** 10.3389/fcimb.2026.1819326

**Published:** 2026-05-29

**Authors:** Edwin J.A. Veldhuizen, Sasja T. van der Velden, David Alons, Lindert Benedictus, Indy van der Drift, Irene S. Ludwig, Danique E. Schreutelkamp, Marc M.S.M. Wösten

**Affiliations:** 1 Department Biomolecular Health Sciences, Utrecht University, Utrecht, Netherlands; 2 Department Population Health Sciences, Utrecht University, Utrecht, Netherlands

**Keywords:** alternative to antibiotic, antimicrobial peptide, cathelicidins, d-amino acids, host defense peptides, infection, mastitis, *Staphylococcus aureus*

## Abstract

**Introduction:**

To effectively treat bacterial diseases caused by antibiotic-resistant strains, new antimicrobial agents are needed to overcome this resistance. This study explored whether the combination of the antimicrobial peptide CATH-2 with positively charged D-amino acids can be used as a potential alternative to antibiotics for treatment of mastitis in cows.

**Methods:**

Antibacterial assays and bacterial growth kinetics were assessed in the presence of CATH-2 combined with either D-arginine or D-lysine. Synergistic interactions between the two components were evaluated using checkerboard assays. Cytotoxicity of the tested compounds was determined through WST, LDH release, and trypan blue exclusion assays on multiple mammalian cell lines, including bovine udder epithelial cells.

**Results:**

The combination of CATH-2 and D-arginine showed strong synergistic antibacterial activity against Gram-positive bacteria, including all tested multidrug-resistant clinical *Staphylococcus aureus* isolates, in both LB medium (FIC = 0.37) and fresh raw milk (FIC < 0.12). No detectable cytotoxic effects on bovine udder epithelial cells or other epithelial cell lines were observed at concentrations that were effective against bacterial growth.

**Conclusions:**

This novel combination of an antimicrobial peptide with a positively charged D-amino acid offers promising potential for treating bacterial infections, particularly those caused by antibiotic-resistant strains.

## Introduction

The overuse and misuse of antibiotics in human and animal health and agriculture have contributed to the rise of antibiotic-resistant bacterial strains, creating a global issue that requires urgent action and innovative solutions (Ho et al., 2025). This crisis makes infections harder to treat, puts modern medicine at risk, and has led to millions of deaths globally. Preferably, alternatives to conventional antibiotics should act through mechanisms distinct from those of classic antibiotics, thereby avoiding cross-resistance, and should not themselves promote the development of bacterial resistance.

A potential alternative for the use of antibiotics is the use of antimicrobial peptides (AMPs). These small cationic proteins have broad spectrum antibacterial activity against both Gram-positive and Gram-negative bacteria (Ageitos et al., 2017). Most AMPs act by binding to the negatively charged bacterial membrane leading to permeabilization of this membrane and subsequent leakage of the cytosolic content, eventually killing the bacterium (Mookherjee et al., 2020). The AMP CATH-2, is a 26 amino-acid AMP from chickens which has shown high antibacterial activity against a number of bacteria (van Dijk et al., 2009). Importantly, it was described that long term exposure of bacterial strains to low levels of CATH-2 did not lead to development of bacterial resistance, unlike common antibiotics (Veldhuizen et al., 2013). There are however some concerns regarding the therapeutic use of AMPs, under physiological conditions they often lose their activity as they are susceptible to proteases (Rima et al., 2021). Incorporation of D-amino acids into AMPs has demonstrated to improve their pharmacokinetic properties and therapeutic effectiveness (Li et al., 2016).

D-amino acids, the enantiomeric counterparts of L-amino acids, might be another potential alternative for antibiotics. They have been long believed to play only a minor role in bacteria as part of the peptidoglycan layer of the bacterial cell wall. Nowadays we know that they are also found in animals and humans where they play a major role in several physiological processes and functions, such as signaling molecules in neurotransmission (Bastings et al., 2019) and hormone secretion (Homma, 2007). In 2009, it was shown that several bacteria secrete millimolar concentrations of D-amino acids into their environment (Lam et al., 2009). These secreted D-amino acids can have regulatory roles, including inhibition of competing bacteria in polymicrobial communities, such as biolfilms (Leiman et al., 2013). For example, *Vibrio cholerae* uses D-arginine as a toxic extracellular effector influencing the growth and morphogenesis of competing microbes (Alvarez et al., 2018).

Mastitis in cows is an inflammatory condition of the udder tissue, commonly caused by bacterial infection, and is one of the most significant diseases in dairy cows, compromising welfare and leading economic losses impact on dairy farms (Goulart and Mellata, 2022; Rainard et al., 2018). In the Netherlands the financial losses for the dairy industry are on average €240/lactating cow per year (Urakawa et al., 2022; van Soest et al., 2016). The disease can be classified into clinical and subclinical forms, with clinical mastitis presenting visible signs such as swelling, redness, and abnormal milk, while subclinical mastitis often remains undetected but can lead to substantial losses in milk yield and quality. The main pathogens responsible for bovine mastitis are the Gram-positive bacteria *Staphylococcus aureus, Streptococcus uberis* and *Streptococccus agalactiae* and the Gram-negative bacterium *Escherichia coli* (Goulart and Mellata, 2022). Antibiotic treatment is the current preferred treatment option, but antibiotic resistance is a growing concern in the treatment of mastitis in dairy cows (Inamullah et al., 2025), complicating management strategies and threatening animal welfare.

This study aimed to investigate whether the antimicrobial activity of CATH-2 could be enhanced by specific D-amino acids, offering a potential novel treatment for mastitis caused by antibiotic-resistant *S. aureus*, *E. coli, S. uberis and S. agalactiae* bacteria.

## Methods

### Bacterial strains tissue culture cells and media

*S. aureus* WKZ2 (Jansen et al., 2006) and USA300 (Diep et al., 2006) are multi-resistant strains, *S. aureus* Saujes and JHs139 were isolated from a cow with subclinical mastitis, *S. aureus* JHs58 was isolated from a cow with clinical mastitis (Hoekstra et al., 2018), while *S. aureus* ATCC25923 is a clinical isolate from a human wound infection. *E. coli* strain 1120–2 was isolated from a clinical mastitis case (isolated at our Population Health Sciences department). *S. uberis* 233 h4c1 and *S. agalactiae* are two clinal mastitis isolates (Swanson et al., 2009). *S. aureus* and *E. coli* were cultured on Luria Broth Agar plates (Thermo Scientific Oxoid, Basingstoke, UK), while *S. uberis* and *S. agalactiae* were cultured on Sheep blood agar plates. A single colony was picked from all strains for o/n growth in Luria Broth (LB, BioTRADING Benelux B.V., Mijdrecht, The Netherlands). HeLa cells (Rodriguez et al., 1999) and Caco-2 cells (ATCC-HTB-37) were grown in 25 cm^2^ flasks in Dulbecco’s modified Eagle’s medium (DMEM) containing 10% fetal calf serum (FCS) at 37˚C and 10% CO_2_. The *parenchyme sécrétoire* (PS) cell line (Roussel et al., 2015), a spontaneously immortalized cell line originating from cultured primary bovine mammary epithelial cells (bMECs), was cultured in T25 flasks containing advanced DMEM Media (RSM, Gibco, Thermo Fisher Scientific, MA, USA) including 10 ng/ml Insulin like growth factor (IGF-1), 5 ng/ml Fibroblast growth factor (FGF), 5 ng/ml Epidermal growth factor (EGF), 0.01% Hydrocortisone, 2% Hepes and 1% Glutamax. The cell line was cultured at 37 °C with a humidified atmosphere of 10% CO_2_.

### Peptide synthesis

Chicken cathelicidin 2 (CATH-2), RFGRFLRKIRRFRPKVTITIQGSARF, was synthesized by China Peptides (Shanghai, China) using Fmoc-chemistry. The peptide was purified by reverse phase high-performance liquid chromatography to a purity of >95%.

### Chemicals

D-amino acids used in this study were purchased from Sigma-Aldrich Inc (Saint Louis, Missouri, US).

### Growth curves

Growth curves of *S. aureus*, *E. coli, S. uberis and S. agalactiae* were recorded on a BioTek Synergy HTX Multimode Reader. In a standard assay 200 µl of bacteria with a final density of 1x10^6^ CFU/ml was added to a flatbottom 96 wells plate in the presence (or absence) of varying concentrations of CATH-2 and D-amino acids. The OD_600_ was measured every 10 min for 24 h at 37 °C under continuous shaking.

### Colony count assays under aerobic conditions

*S. aureus* and *E. coli* cultures were grown from a fresh overnight culture to logarithmic phase in LB under constant shaking of 160 RPM at 37 °C. From logarithmic phase the bacteria were diluted to a density of 1x10^6^ CFU/ml. To 25 µl bacterial solution 25 µl LB was added containing CATH-2 (0-40 µM) and D-Arginine (0–20 mM) in a polypropylene round bottom 96 wells plate. After 3 h incubation at 37 °C, serial 10-fold dilutions were prepared, which were plated on Tryptone Soy Agar (TSA) plates. After o/n incubation at 37 °C, viable bacteria were counted.

### Colony count assays under anaerobic conditions

Overnight cultures of *S. aureus* and *E. coli* were grown anaerobically under constant shaking of 160 RPM at 37 °C. Bacteria were diluted to a concentration of 1x10^6^ CFU/ml in LB and incubated with 20 mM D-Arg with or without 5 µM CATH-2 under anaerobic conditions. At various times during the 3 h incubation, samples were taken, serially diluted in 10-fold increments and plated on TSA plates. After o/n incubation at 37 °C, viable bacteria were counted.

### Determination of synergy between CATH-2 and D-Arg

Checkerboard dilution assays were performed both in LB and raw milk to determine possible synergy between CATH-2 and D-Arg. For the assays in LB, 1x10^6^ CFU/ml *S. aureus*, *S. uberis* or *S. agalactiae* were treated in 96 wells plates with all combinations (using twofold dilutions) of CATH-2 (0-80 µM) and D-Arg (0–160 mM). Visual determination of growth was performed after 24 h incubation at 37 °C. In raw milk a similar set-up was used but growth was determined by plating out the incubation mixtures on TSA, because visual determination of growth is impossible due to the turbidity of the milk. Separate MBC determinations for D-Arg only and CATH-2 only in milk were performed because they were outside of the used concentrations in the checkerboard assays. Synergy was determined by calculating the fractional inhibitory concentration index (FICI) = MIC of drug A in combination/MIC of drug A alone + MIC of drug B in combination/MIC of drug B alone. In milk the MBC of CATH-2 was 320 µM, while D-Arg did not obtain an MBC at 320 mM, but 320 mM was used for FICI calculations. The calculated FICI was interpreted as synergistic for FICI <0.5, additive: 0.5< FICI<1; indifferent (1< FICI <4.0), or antagonistic FICI> 4.

### Toxicity: WST-1 assay

To study the effect of CATH-2 and D-Arg on cell viability, Hela cells and Caco-2 cells were seeded at a density of 2x10^4^ cells per well in 96 well plates. These cells were subjected to various concentrations of CATH-2 and D-Arg in growth medium for 4 h. Following the incubation period, the culture medium was substituted with 100 μL of new medium solution containing 10% water-soluble tetrazolium 1 (WST-1) (Roche, Basel, Switzerland). Subsequent colorimetric analysis was conducted after a 10−15 min incubation period at 450 nm using a FLUOstar Omega microplate reader (BMG Labtech GmbH, Ortenberg, Germany). Cell viability was then quantified as a percentage, with 100% indicating the viability of untreated cells.

### Toxicity: LDH leakage assay

Toxicity of CATH-2 and D-Arg toward Hela and Caco-2 cells was determined by the LDH release assay, as described previously. In short, cells were incubated with combinations of CATH-2 and D-Arg for 4 h and cytotoxicity was evaluated by measuring the proportion of LDH released into the supernatant. Cytotoxicity was expressed relative to the maximum LDH release observed in cells lysed with Triton X-100 detergent. This assessment was performed using the Cyto Tox 96 nonradioactive cytotoxicity kit (Promega), following the manufacturer’s instructions.

### Toxicity assay with PS (bMEC) cells

The PS cells were seeded at a density of 2x10^4^ cells per well in a flat-bottom polystyrene 96-well plate and o/n incubated at 37 °C and 10% CO_2_, which allowed attachment and proliferation of the cells, thereby forming a monolayer of cells. The cells were subjected to various concentrations of CATH-2 (0-40 µM) and D-Arg (0–40 mM) dissolved in day-fresh raw milk for a duration of 1 h and 4 h. All solutions were prepared such as to contain 85% raw milk and 15% water. As a positive control, cells were exposed to methanol for a duration of 1 min. After incubation, the cells were washed two times with PBS, followed by a 5 min incubation with 1:1 trypan blue. The cells were washed an additional two times with PBS. To ensure accurate quantification, four non-overlapping images were acquired per well and the stained and unstained cells were counted.

### Antibacterial activity of CATH-2 and D-Arg, D-Lys or D-ser in milk

In order to determine the antibacterial activity of CATH-2 and D-Arg in a relevant medium, colony count assays were performed in raw milk (Farm De Tolakker of the Veterinary Faculty, Utrecht). *S. aureus* cultures were grown to logarithmic phase as described above. Subsequently, fresh, bulk tank milk, 4^0^C was spiked with 1x10^6^ CFU/ml *S. aureus* and subsequently treated with CATH-2 and D-Arg, D-Lys or D-Ser from stock solutions in milliQ, (resulting in a final percentage of 85% milk, 0.2% LB and 14.8% water) and incubated at 37 °C. At varying time points small aliquots were taken from the incubation mixture, serially diluted and plated on TSA plates. These were incubated for 24 h at 37 °C and viable bacteria were counted.

### Isothermal titration calorimetry

The experiments were performed on a NanoITC (TA Instruments, New Castle, USA). All components (CATH-2, *Pseudomonas aeruginosa* LPS, and D-Arg) were diluted to the indicated concentrations in 75% (v:v) phosphate-buffered saline (PBS). The 164 µl chamber was filled with 100 µM CATH-2 and the syringe with 1 mM D-Arg. Titrations were incremental with 2 µl injections at 300 s intervals. Experiments were performed at 37 °C. Control experiments were performed titrating 1 mM D-Arg into a 75% PBS solution in the chamber (negative control) and titrating 200 µM CATH-2 into 10 µM LPS (positive binding control).

### Statistical analysis

Statistical analysis was performed using Graphpad Prism 10 (GraphPad, San Diego, CA). Data is expressed as mean with SEM. Results of growth curve and survival test assays were analyzed by measuring the area under the curve, followed by one-way Anova using Dunnett’s multiple comparisons test. Toxicity data were analyzed using a one-way Anova. P-value of <0.05 was considered statistically significant. (*P<0.05, **P<0.01, ***P<0.001, ****P<0.0001).

## Results

### Antibacterial activity of D-Amino acids

**Figure 1:**
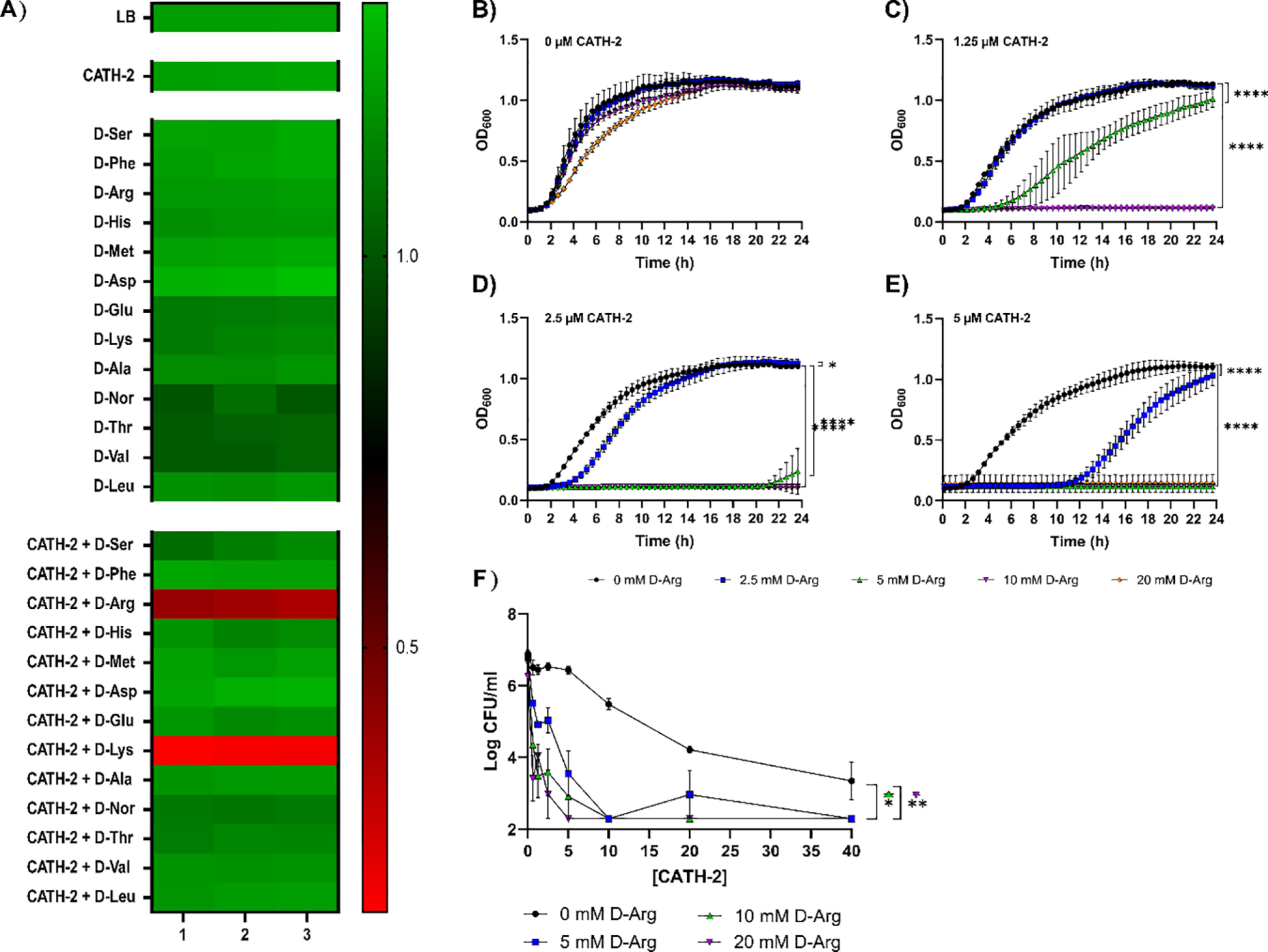
Antibacterial effect of D-Arginine and CATH-2. **(A)** Growth curves of S. aureus WKZ2 were generated in the presence or absence of 2 µM CATH-2 and 20 mM D-AA in LB at 37 °C. The mean final OD600 values after 12 hours from 3 replicates of 3 independent experiments are schematically shown in a heatmap. Growth curves of S. aureus WKZ were generated with different concentrations of D-Arg as indicated with **(B)** 0 µM CATH-2; **(C)** 1.25 µM CATH-2; **(D)** 2.5 µM CATH-2 and **(E)** 5 µM CATH-2 at 37 °C and growth was determined by measuring OD_600_ every 10 min. Data are represented as mean ± SEM of three independent experiments. P-value, *P < 0.05, **P < 0.01, ***P < 0.001, ****P < 0.0001. **(F)** Bacterial viability was determined after S. aureus WKZ2 was incubated for 3 h with different concentrations of D-Arg and CATH-2. Shown are the average + SEM of 3 independent experiments. P-value, *P < 0.05, **P < 0.01.

The antimicrobial peptide CATH-2 is known to possess antibacterial activity, however the activity of antimicrobial peptides are often strongly dependent on the presence of proteases and divalent cations (Mookherjee et al., 2020). As certain D-Amino Acids (D-AAs) are known to enhance the effectiveness of antibiotics (Yang et al., 2024), we sought to explore whether D-AAs could also boost the efficacy of CATH-2. Therefore, we tested whether all soluble D-AAs in combination with or without sublethal concentrations of CATH-2 possess antimicrobial properties against multi-antibiotic resistant isolate *S. aureus* strain WKZ2. First the final optical density of *S. aureus* cultures containing sublethal 2 µM CATH-2 with or without 20 mM D-AA was measured. No bacterial growth defect was observed when only 2 µM CATH-2 was added (**Figure 1A**). When D-amino acids were added on their own, only D-Nor, D-Thr and D-Val slightly reduced the final optical density of the culture compared to LB alone. However, when CATH-2 was combined with either D-Lys or D-Arg a strong reduction of the optical density, *i.e.* bacterial growth inhibition was observed, indicating that CATH-2 together with these two D-amino acids are potentially synergistic antibacterial combinations.

Next, growth curves were generated of *S. aureus* JHs58 (a clinical mastitis isolate) in the presence of 0–40 mM D-Arg, D-Lys or D-Ser (as negative control). At concentrations of 20 mM and 40 mM D-Arg and to a lower extend with D-Lys, growth of *S. aureus* was inhibited as seen by the extended lag time (**Supplementary Figure S1**). However, this inhibitory effect was absent at lower concentrations of these D-amino acids. D-Ser showed no impact on *S. aureus* growth at any concentration tested.

### Antibacterial activity of CATH-2 in combination with D-Arg or D-Lys

To address how CATH-2 combined with D-Arg or D-Lys influence the growth of *S. aureus* we generated growth curves using varying concentrations CATH-2 (0-5 µM) and D-Arg or D-Lys (0 to 20 mM) (**Figures 1B–E**). We observed that by increasing the amount of D-Arg or D-Lys in the presence of CATH-2, not only the lag phase extended, but also the slope and to a lower extend the final optical density was reduced. This suggest that it takes longer before the bacteria have been adapted to the culture medium and that uptake of nutrients is slower in the presence of both components. No growth was observed with 2.5 µM CATH-2 with 20 mM D-Arg or 5 µM CATH-2 with 5-, 10- or 20 mM D-Arg (**Figures 1D, E**). Similar results were obtained for CATH-2 and D-Lys (**Supplementary Figure S2**).

To explore the effects of combined treatment with CATH-2 and D-Arg on the survival of *S. aureus*, colony count assays were performed using different concentrations of D-Arg (0–20 mM) and CATH-2 (0-40 µM). As depicted in **Figure 1F**, CATH-2 alone was relatively weakly active against *S. aureus*. However, addition of 5 mM or higher concentrations of D-Arg had a strong concentration-dependent positive effect on the antibacterial activity of CATH-2 leading to MBC values of 10 µM and lower.

### All *S. aureus* strains are sensitive to the CATH-2/D-Arg combination

**Figure 2:**
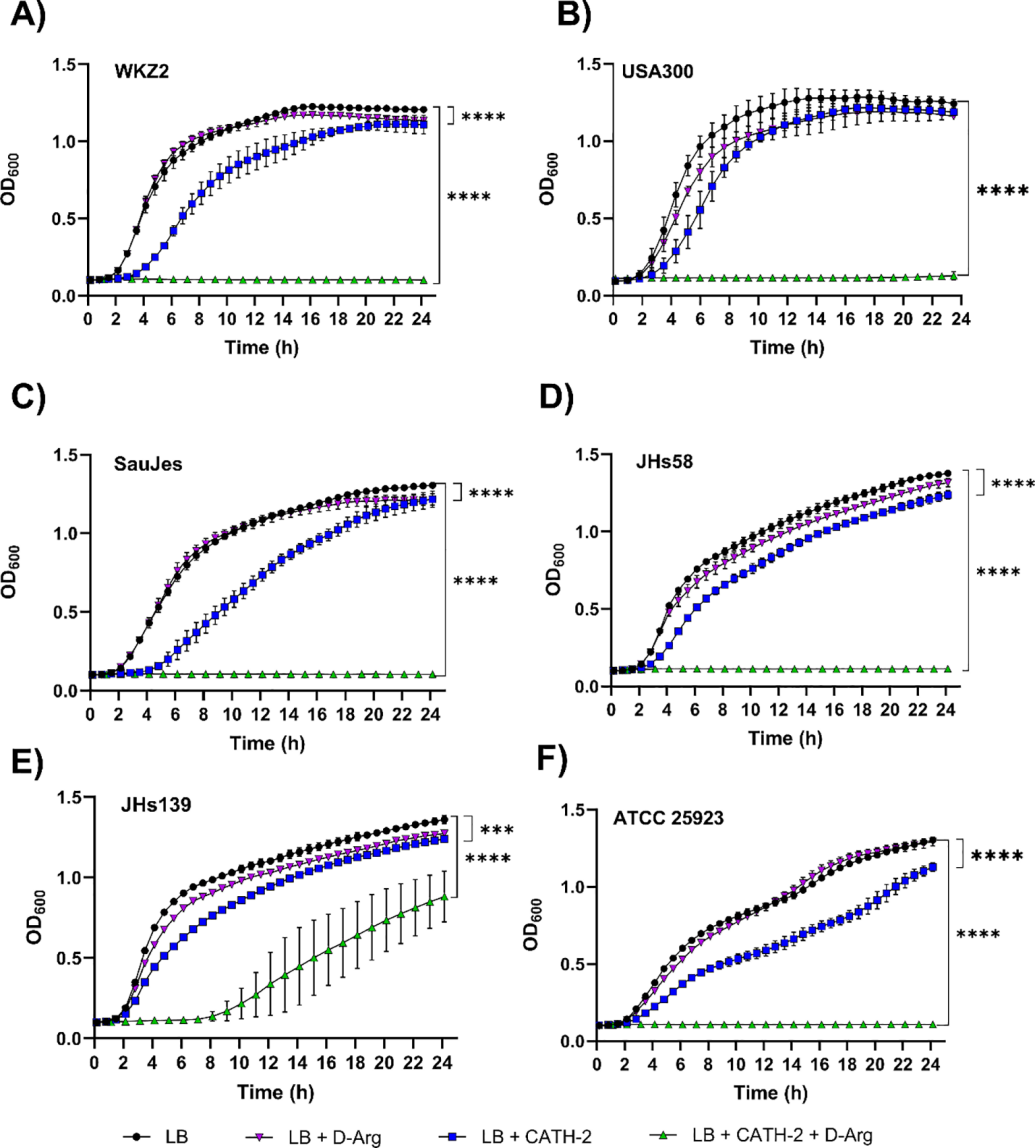
Effect of CATH-2 and D-Arg on growth of six S. aureus strains. S. aureus strains **(A)** S. aureus WKZ2, **(B)** S. aureus USA300, **(C)** S. aureus SauJes, **(D)** S. aureus JHs58, **(E)** S. aureus JHs139 and **(F)** S. aureus ATCC 25913 were grown with or without 5 µM CATH-2 and/or 5 mM D-Arg at 37 °C. Growth was determined by measuring OD_600_ every 10 min. Growth curves are represented as mean ± SEM of three independent experiments. P-value, ***P < 0.001, ****P < 0.0001.

**Figure 3:**
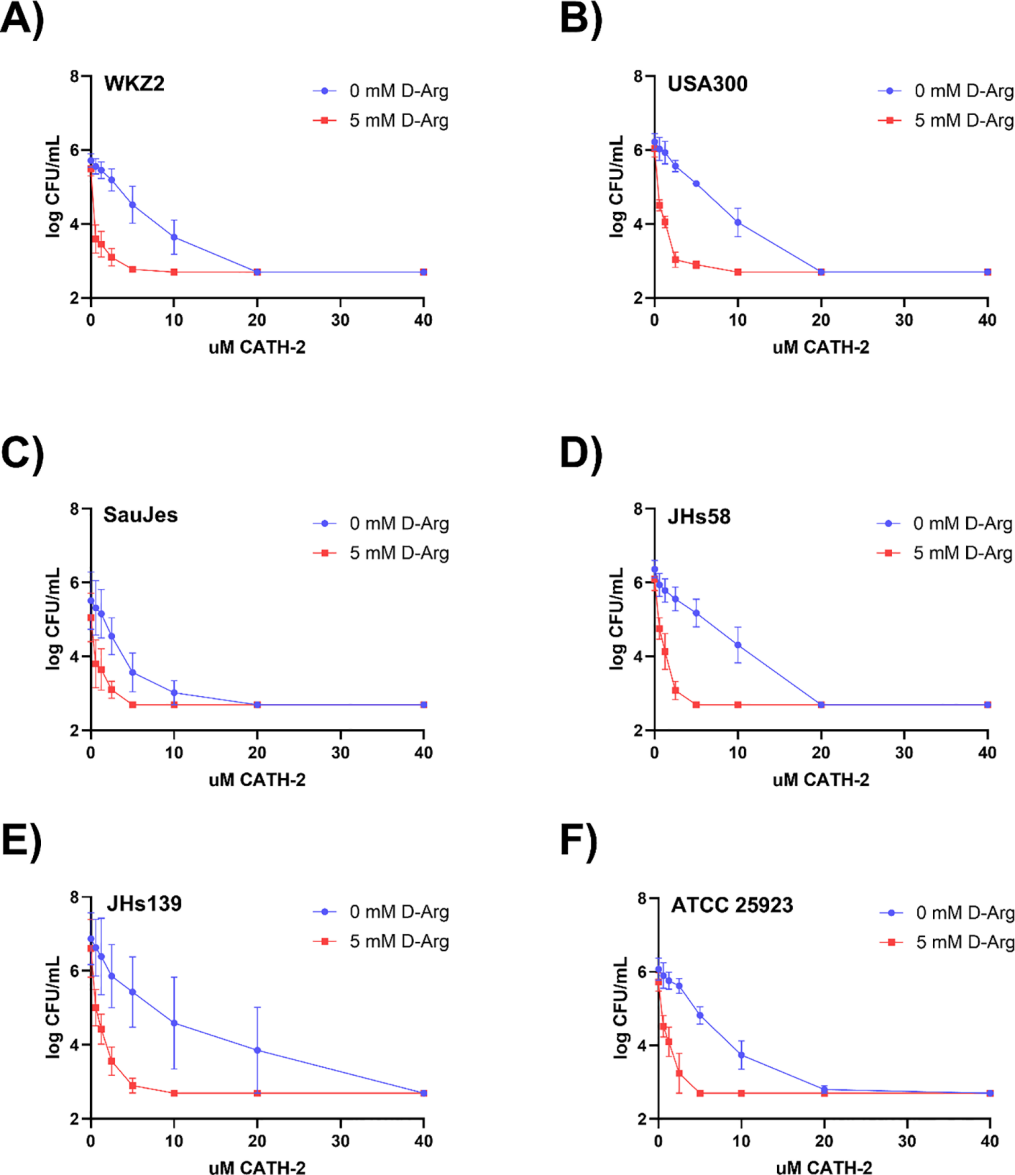
CATH-2/D-Arg is active against multiple S. aureus strains. Colony count assays were performed on 6 S. aureus strain comparing the effect of CATH-2 alone and in combination with 5 mM D-Arg. **(A)** S. aureus USA300, **(B)** S. aureus ATCC 25913, **(C)** S. aureus SauJes **(D)** S. aureus JHs58, **(E)** S. aureus JHs139 and **(F)** *S. aureus* ATCC 25923. Bacteria were incubated with CATH-2 +/- 5 mM D-Arg for 3 h at 37 °C Subsequently, bacterial viability was determined by plating out on TSA plates. Shown are the average + SEM of 3 independent experiments.

To rule out strain-specificity of the observed antibacterial effect of the CATH-2/D- Arg combinations, we subsequently tested six different *S. aureus* strains. The strains included two multi-resistant *S. aureus* strains (WKZ2 and USA300), two strains isolated from a cow with subclinical mastitis (JHs139 and SauJes), one from a cow with clinical mastitis (JHs58) and the ATCC 25923 strain. As shown in **Figure 2** there was hardly any difference between the growth curves of the strains. All strains were able to grow in the presence of 5 mM D-Arg or 5 µM CATH-2 separately, however when these compounds were both present growth of all strains was completely inhibited, except for strain JHs139, which started to grow after an 8 h long lag phase (**Figure 2E**). Similar effects were seen in bacterial viability assays (**Figure 3**) where no viable bacteria were detected for all strains when 10 µM CATH-2 and 5 mM D-Arg was present and very strong reductions in viable bacteria at a one-step lower concentration of 5 µM CATH-2 and 5 mM D-Arg (the conditions similar to **Figure 2**).

### Antibacterial activity of the CATH-2/D-Arg combination against other mastitis causing bacteria

To investigate whether the CATH-2/D-Arg combination also had an effect on other mastitis causing bacteria like *E. coli*, *S. uberis* and *S. agalactiae*, we generated growth curves using varying concentrations of CATH-2 (0-5 µM) and D-Arg (0 to 20 mM) (**Supplementary Figures S3**-**S5**). In contrast to *S. aureus*, increasing the concentration of CATH-2 alone strongly extended the lag phase of *E. coli* (**Supplementary Figure S3**). The addition of D-Arg alone hardly affected the growth of *E. coli* and also in combination with 1.25 µM CATH-2, addition of D-Arg resulted only in a minor additional effect on growth. At higher CATH-2 concentrations the peptide’s effect alone was already (almost) completely blocking bacterial growth as no additional effects of D-Arg could be detected.

Both *S. uberis* and *S. agalactiae* were sensitive for D-Arg alone (**Supplementary Figures S4A**, **S5A**), contrary to *S. aureus* and *E. coli.* While increasing D-Arg concentrations resulted in a similar lag-phase but clearly lower final optical density for *S. uberis*. For *S. agalactiae* the lag phase was strongly extended in the presence of D-Arg. Both strains were also very sensitive to CATH-2. In the presence of D-Arg and 2.5 µM CATH-2 both strains were unable to grow (**Supplementary Figures 4C**, **S5C**), showing a higher sensitivity for these bacteria to CATH-2/D-Arg then *S. aureus*.

### Detection of synergy between CATH-2 and D-Arg

**Figure 4:**
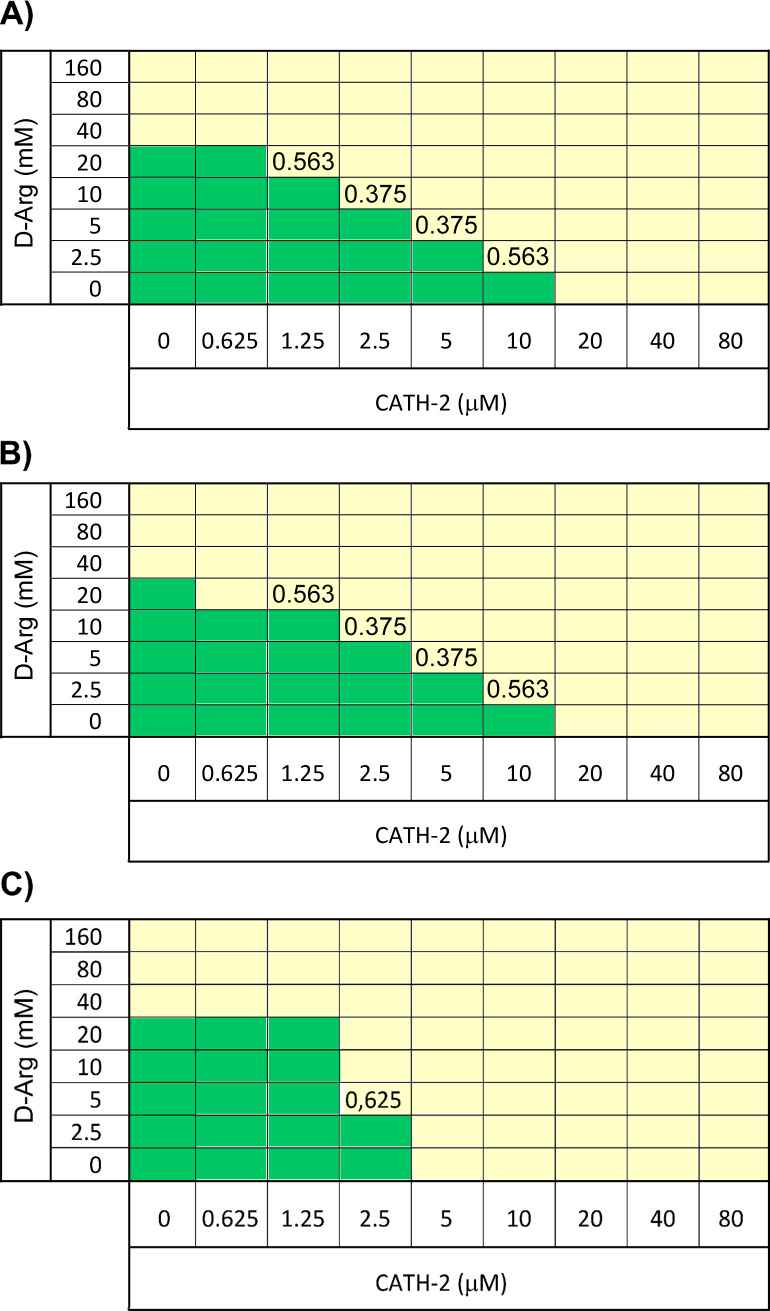
Checkerboard assay shows synergistic combinations of CATH-2 and D-Arg. *S. aureus JHs58* **(A)***, S. uberis* 233 h4c1 **(B)** *and S. agalactiae* **(C)** were incubated with different ratios of CATH-2 (0-80 µM) and D-Arg (0–160 mM) in 96 wells plates. After 24 h growth in each well was visually assessed and FIC indices were calculated for each combination where no growth was visible.

To investigate whether CATH-2 and D-Arg exhibit a synergistic antibacterial effect against *S. aureus*, *S. uberis* and *S. agalactiae*, checkerboard assays in LB were performed (**Figure 4**). From this figure it is seen that the minimum inhibitory concentration of D-Arg is 40 mM for all three bacteria while CATH-2 prevented growth of *S. aureus*, *S. uberis* and *S. agalactiae* at 20, 20, and 5 µM, respectively. Potential synergy was determined by calculating the fractional inhibitory concentration index (FICI), of the CATH-2/D-Arg combinations, FIC indices ranged from 0.375 – 0.625 for specific ratios of CATH-2 and D-Arg. For example, the combination of 5 µM CATH-2 and 5 mM D-Arg completely blocked *S. aureus* growth and resulted in a FIC index of 0.375, while other ratios such as 1.25 µM CATH-2 + 20 mM D-Arg resulted in a FIC index just above 0.5. These results show that CATH-2 and D-Arg work synergistically against *S. aureus* and *S. uberis* but not against *S. agalactiae*.

### Synergistic activity of D-Arg and CATH-2 under an- vs aerobic conditions

**Figure 5:**
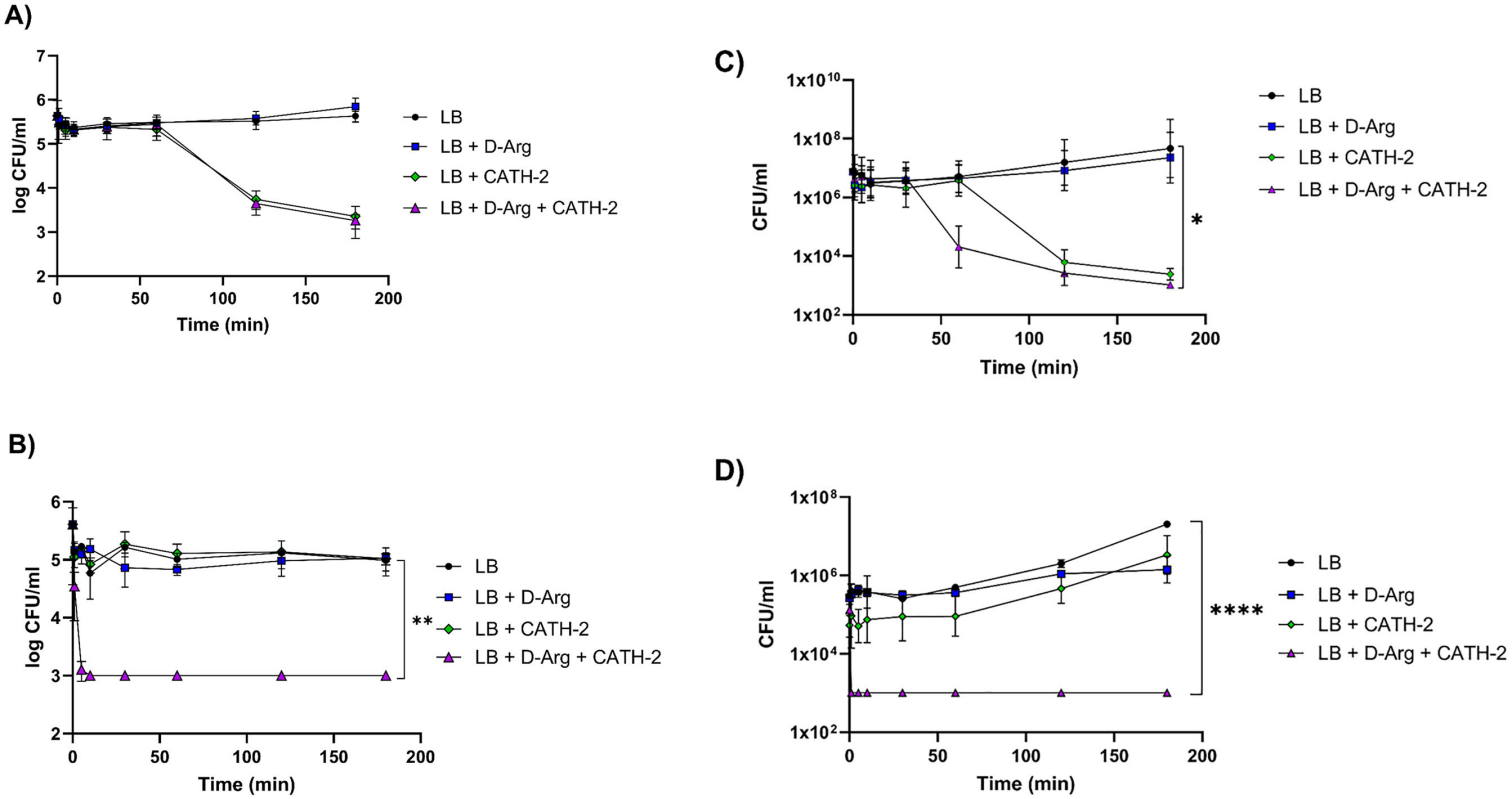
Antibacterial effect of CATH-2 and D-Arg under different oxygen conditions. E. coli **(A, C)** and S. aureus **(B, D)** were incubated under anaerobic **(A, B)** and aerobic **(C, D)** conditions with 5 µM CATH-2 with or without 20 mM D-Arg for 3 h. At the indicated time points samples were taken and 10-fold serial dilution were made and plated onto LB agar plates. After overnight incubation at 37°C the CFigure were counted. Shown are the average + SEM of 3 independent experiments. P-value, *P < 0.05, **P < 0.01, ****P < 0.0001.

Because mastitis leads to a dramatic drop in the oxygen concentration (Mayer et al., 1988) we investigated whether the CATH-2/D-Arg combination also works under anaerobic conditions. For this, *S. aureus* ATCC25923 and *E. coli* were incubated under anaerobic or aerobic conditions with 20 mM D-Arg, with or without 5 µM CATH-2 (**Figure 5**). Under anaerobic as well as aerobic conditions, *E. coli* was highly sensitive to CATH-2 alone, and the combination of CATH-2 and D-Arg resulted in only a minor additional reduction of CFU (**Figures 5A, C**). In contrast to *E. coli*, CATH-2 alone had no effect on the growth of *S. aureus*, but the CATH-2/D-Arg combination killed all bacteria under both oxygen conditions (**Figures 5B, D**). In addition, the kinetics of killing *S. aureus* bacteria was similar for both oxygen conditions.

### Toxicity assays LDH and WST towards Hela and Caco-2 cells

**Figure 6:**
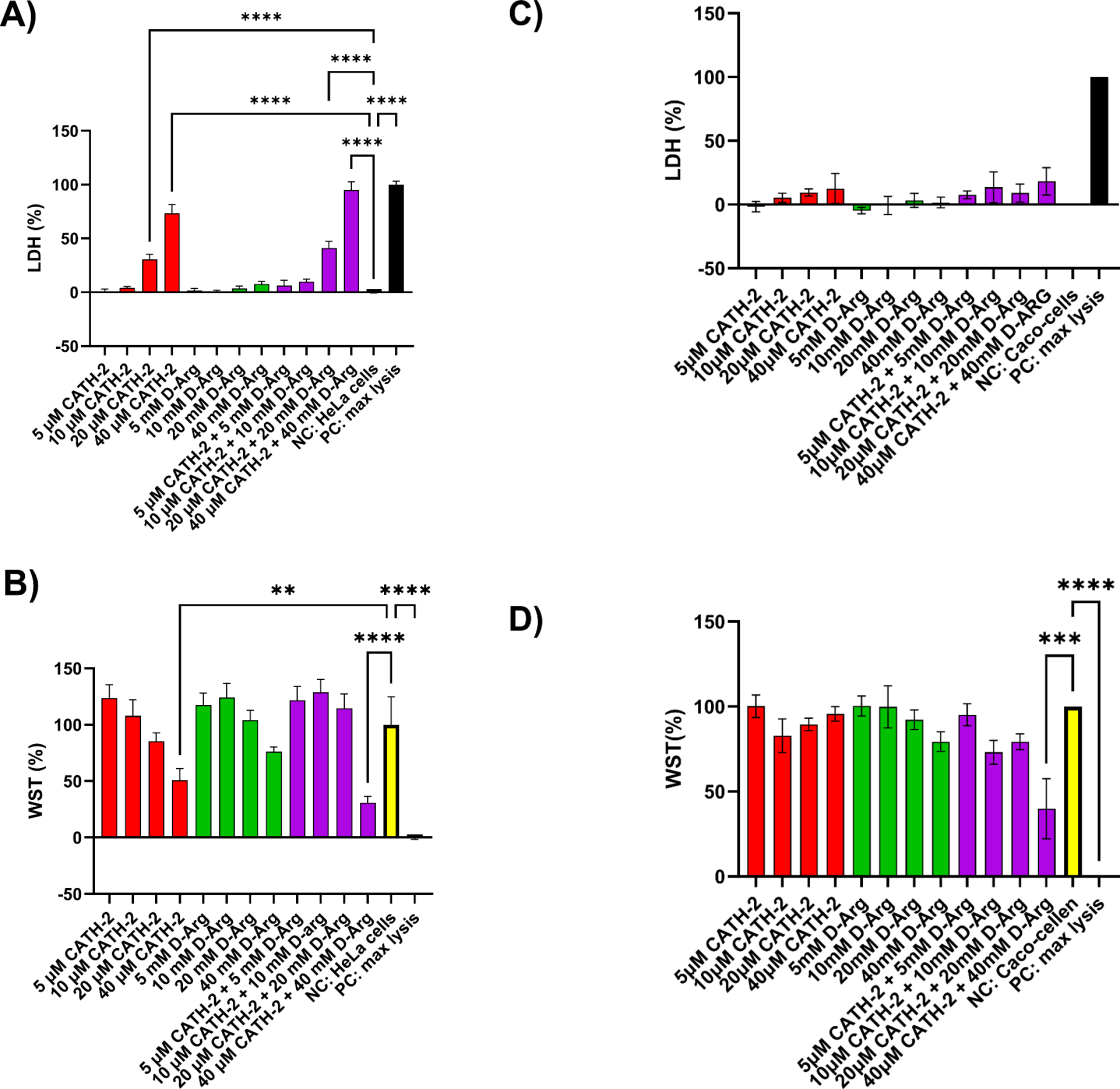
Effect of the CATH-2/D-Arg combination on the cell permeability, and metabolic activity of Hela and Caco cells. Permeability of the Hela cells were investigated by LDH **(A)** and metabolic activity by WST-1 **(B)** assays using different concentrations D-Arg and CATH-2. Permeability of the Caco cells were investigate by LDH **(C)** and metabolic activity by WST-1 **(D)** assays. Shown are the average + SEM of 4 independent experiments. P-value, **P < 0.01, ***P < 0.001, ****P < 0.0001.

To investigate whether the CATH-2/D-Arg combination is toxic for eukaryotic cells, WST and LDH assays were performed on Hela and Caco-2 cells. Cells were incubated for 4 h with different concentrations of CATH-2 and D-Arg. Above 10 µM CATH-2 was toxic for the Hela cells (**Figures 6A, B**), but not for the Caco-2 cells (**Figures 6C, D**). Only the combination 40 µM CATH-2 and 40 mM D-Arg, the highest concentration tested, was to some extend toxic for Caco-2 cells (**Figure 6D**). These results show that D-Arg is not toxic even at the highest concentration tested, but that CATH-2 shows some toxicity above 10 µM for one cell line.

### Toxicity assay with PS (bMEC) cells

**Figure 7:**
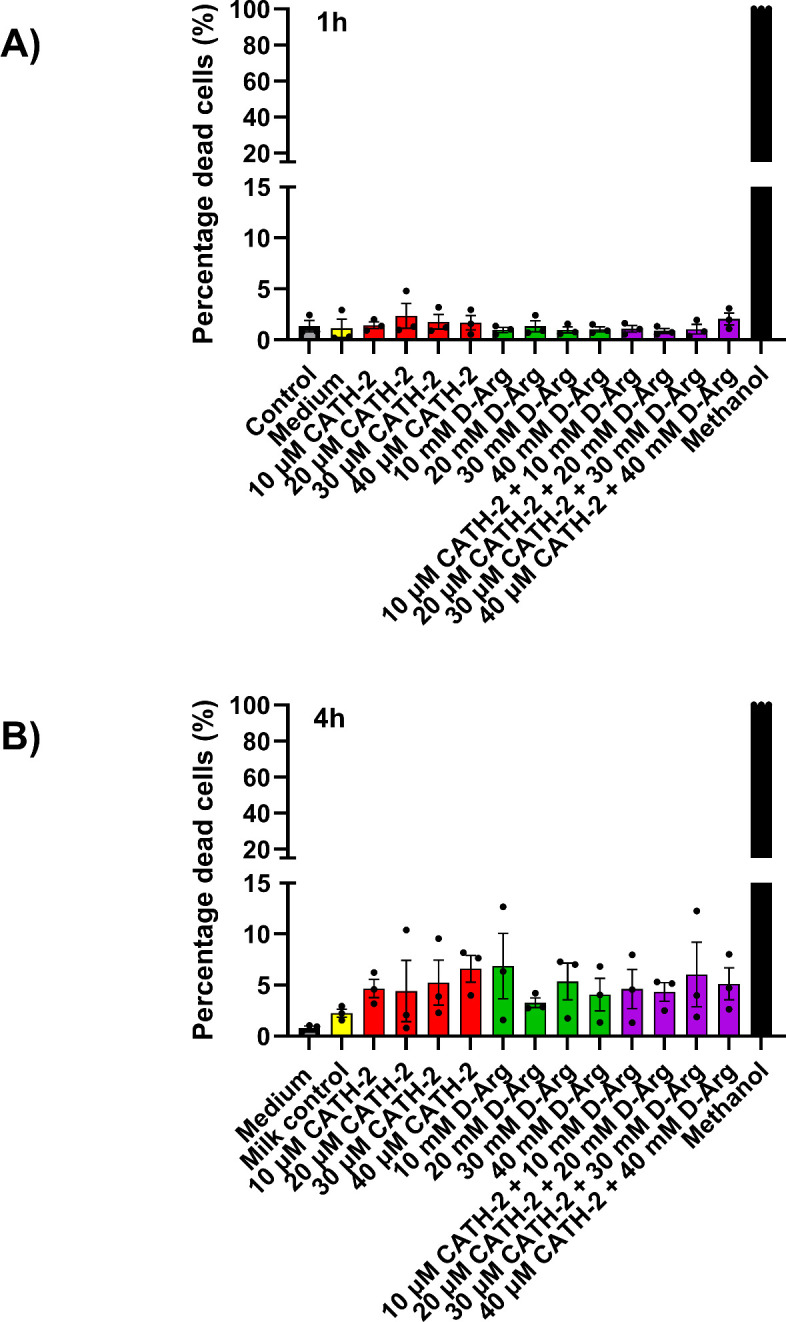
Effect of the CATH-2/D-Arg combination on bovine mammary epithelial (PS) cells in the presence of raw cow milk. PS cells suspended in raw cow milk were exposed to various concentrations CATH-2 (10-40 µM) and D-Arg (10–40 mM) for 1h **(A)** or 4h **(B)**. As a negative control, the cells were only exposed to a solution containing 85% raw milk and 15% water, and as a positive control, the cells were exposed to methanol. After incubation, the cells were stained with trypan blue. Dead cells were counted and expressed as a percentage to the total number of cells. Data are represented as mean + SEM of three independent experiments (n=3).

To obtain a more representative representation of the toxic effects of CATH-2 and D-Arg, the toxicity assay was additionally conducted on bovine mammary epithelial cells (PS cells). They were exposed to multiple concentrations of CATH-2 (0-40 µM) and D-Arg (0–40 mM) dissolved in raw cow milk, for a 1 h and 4 h duration (**Figures 7A, B**, respectively). Because the presence of raw milk interfered with the LDH and WST assays, cell viability was assessed after exposure using a 1:1 ratio of Trypan Blue and RSM. The dead cells were counted and expressed as a percentage relative to the viable cells.

After 1 h of incubation, PS cells treated with CATH-2, D-Arg, or their combination exhibited a maximum toxicity of 2.35%, whereas the negative control (PS cells exposed only to raw cow milk) showed a similar toxicity of 1.25%. After 4 h, the maximum toxicity increased to 6.87%, while the negative control displayed 2.25% toxicity and the positive control (methanol) induced 100% toxicity. No morphological changes of the cells were observed for either incubation periods (data not shown). These results indicate that, under conditions most representative of the *in vivo* situation, the CATH-2/D-Arg combination is non−toxic to bovine mammary epithelial cells.

### Antibacterial activity of CATH-2 and D-Arg in raw milk

**Figure 8:**
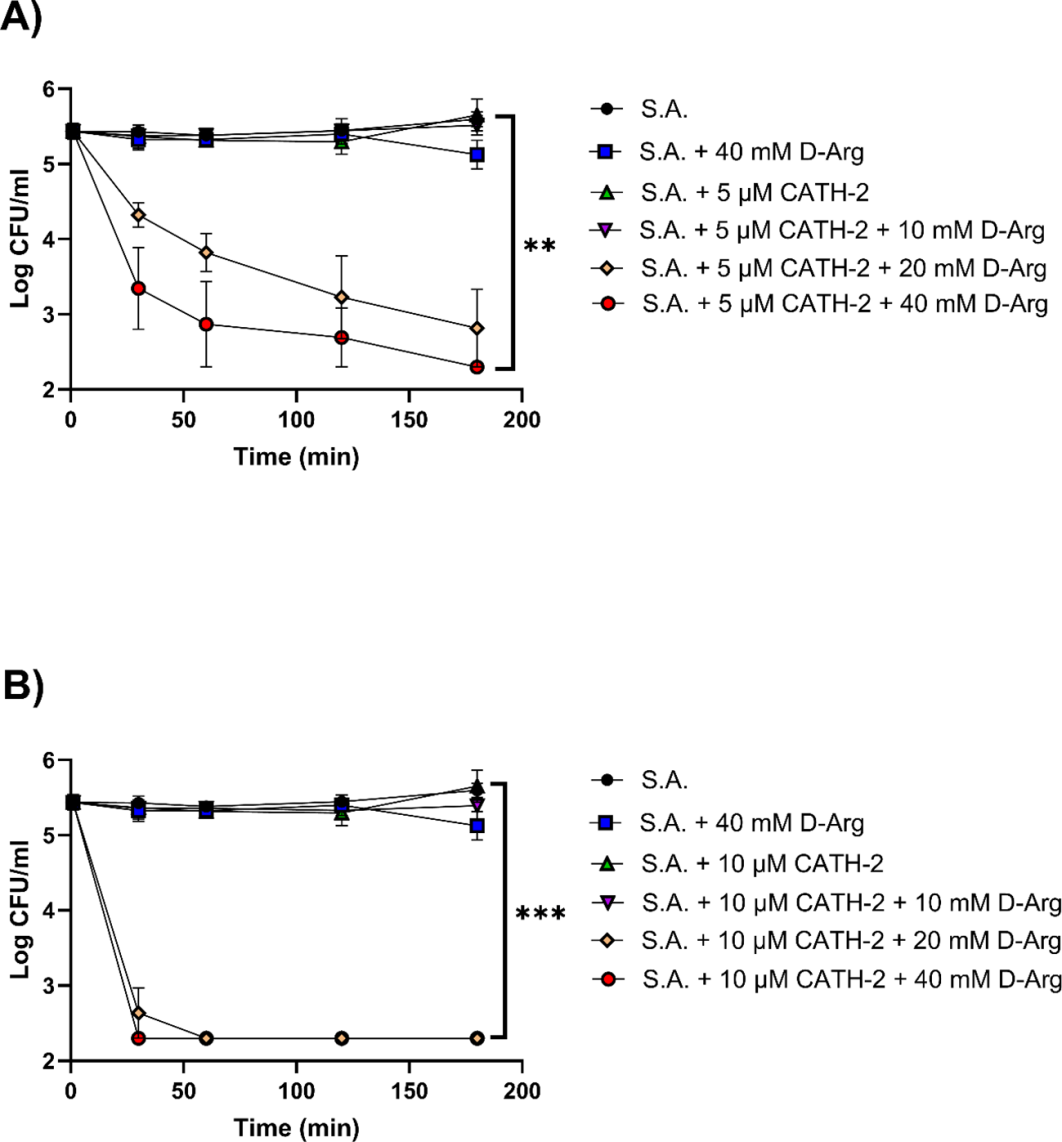
Antibacterial activity of CATH-2 and D-Arg in raw milk. Raw milk was spiked with 1x10^6^ CFU/ml S. aureus JHs58 and incubated with or without **(A)** 5 µM or **(B)** 10 µM CATH-2 with 10, 20 or 40 mM D-Arg. At different time points small samples were taken and surviving bacteria enumerated by plating on TSA. Shown are the average + SEM of 3 independent experiments. P-value, **P < 0.01, ***P < 0.001.

Since milk is the envisioned environment for treatment of mastitis, the antibacterial activity of CATH-2 in the presence or absence of D-Arg was tested in freshly obtained raw milk spiked with *S. aureus* ATCC25923. After treatment, milk was plated and after culture colony-forming units (CFU) were counted to measure bacterial survival. Interestingly, CATH-2 completely lost its antibacterial effect in milk (**Figures 8A, B**, black lines) likely through activity-inhibiting components in the milk. Similarly, 40 mM D-Arg alone had no effect on the survival of the spiked *S. aureus* (**Figure 8A**). However, when both were combined a strong reduction in the number of viable bacteria was seen. At 5 µM CATH-2 and 20 mM D-Arg more than 3-log reduction in CFU/ml was achieved within 3 h, while 10 µM CATH-2 and 20 mM D-Arg led to complete eradication of all bacteria after 1 h (red lines **Figures 8A, B**). Increasing the concentration of D-Arg further to 40 mM showed even faster kinetics of killing. Similar results were obtained when D-Lys was used instead of D-Arg, but D-Ser was unable to lower the *S. aureus* colony forming units (**Supplementary Figure S6**). These results clearly indicate the potential of CATH-2 and D-Arg (or D-Lys) to kill *S. aureus* in raw milk.

### Determination of synergy in raw milk.

**Figure 9:**
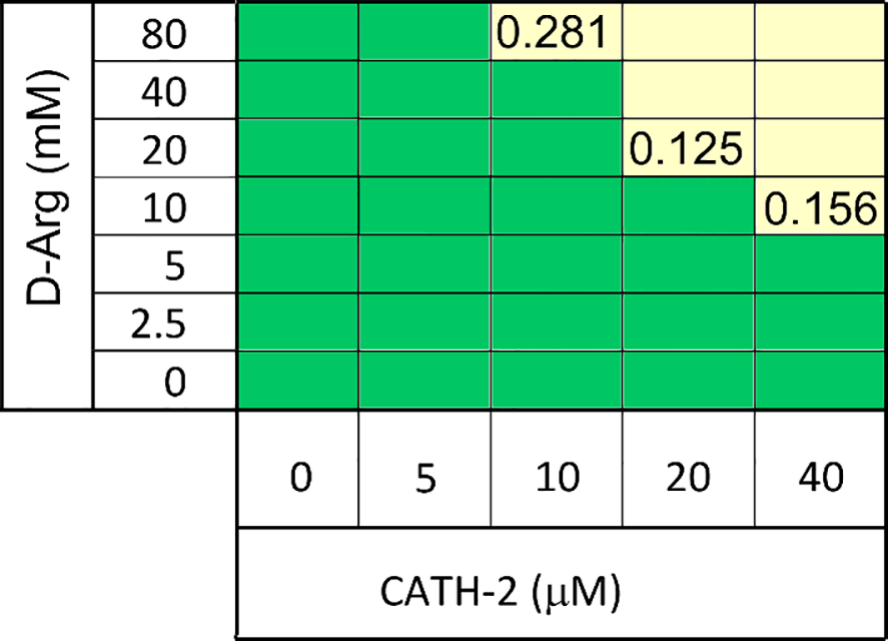
Checkerboard assay of antimicrobial activity of CATH-2 with D-Arg against S. aureus in raw milk. Raw milk was spiked with 1x10^6^ CFU/ml S. aureus JHs58 and incubated with different ratios of CATH-2 (0-40 µM) and D-Arg (0–80 mM) in 96 wells plates. After 24 h, survival of bacteria was assessed by plating out on TSA, and FIC indices were calculated for each combination where no viable bacteria were detected.

To determine whether CATH-2 and D-Arg also exhibit a synergistic effect in raw milk, a checkerboard assay was performed. Since visual end-point registration is impossible in milk each well was plated out on TSA plates to detect viable bacteria. A representative example is shown in **Figure 9** and shows that combinations starting from 10 µM CATH-2 or 10 mM D-Arg resulted in complete killing of bacteria. With an MBC of 320 µM for CATH-2 in milk and no detectable MBC for D-Arg (>320 mM) (data not shown), FICI values were calculated to be between 0.1-0.3, all indicating strong synergy for these components in raw milk.

## Discussion

The rapid emergence of antibiotic-resistant bacteria due to the overuse and misuse of these medications requires an urgent need for new drug development. AMPs have been shown to be active against a broad range of microorganisms, killing both Gram-positive and negative bacteria within a short time, while not inducing resistance (Ageitos et al., 2017; Veldhuizen et al., 2013). However, under physiological conditions they often lose their activity (Rima et al., 2021). Here we report that the positively charged D-amino acids D-Arginine and D-Lysine work synergistically with the antimicrobial peptide CATH-2 against the Gram-positive mastitis causing bacteria *S. aureus*, and *S. uberis.* Alone these compounds have hardly any antibacterial effect, however, combined they effectively kill these bacteria in raw cow milk.

As certain D-AAs are known to enhance the effectiveness of antibiotics (Yang et al., 2024), we sought to explore whether the addition of D-AAs could also boost the efficacy of the antimicrobial peptide CATH-2. All soluble D-amino acids at a concentration of 20 mM had no influence on the growth of *S. aureus* (**Figure 1**), as also observed by other investigators (Alvarez et al., 2018). Also, no growth inhibition was observed when only 2 µM CATH-2 was added confirming our previous results (Veldhuizen et al., 2013). For most D-amino acids, the combination with CATH-2 did not result in growth inhibition. However, a strong growth inhibition was seen when we combined the D-amino acids, D-Lysine or D-arginine with CATH-2. These combinations appear to work synergistically against the mastitis causing bacteria *S. aureus* and *S. uberis* and work well against *S. agalactiae* (**Figure 4**).

Mastitis is considered the costliest disease on dairy farms. Antibiotic treatment of mastitis cases is globally still the standard approach, both for acute cases during lactation using short acting formulations and chronic cases during the dry period using long acting formulations. Intramammary antibiotic therapy is generally recommended only for infections caused by Gram-positive bacteria such as *S. aureus* and *S. agalactiae* (Lago et al., 2011). In contrast, most Gram-negative infections are cleared by the cow’s own immune system (Pyorala et al., 1994). However, in practice the causative agent is often not known and alle mastitis cases meeting specific criteria are treated. Alongside antibiotics, Non-Steroidal Anti-Inflammatory Drugs (NSAIDs) are widely used to reduce inflammation, pain, and fever associated with mastitis. NSAIDs can improve cure rates, but they are not antibacterial and therefore must be used in adjunction to antibiotics (Muzammil et al., 2022). Many mastitis-associated pathogens have become resistant to common antibiotics (Li et al., 2023; Muzammil et al., 2022). Bacteriophage therapy might be promising, however the CRISPR-Cas system which is widely distributed in bacteria can interfere by defending against specific types of phages (Shivram et al., 2021). Therefore, the combination of the AMP CATH-2 with the positively charged amino acids D-Arginine or D-lysine might be a hopeful combination to allow treatment of mastitis in the future.

Consistent with findings in mice (Navarro et al., 2005), D-Arg did not harm tissue culture cells (**Figures 6**, **7**). In contrast, CATH-2 caused some toxicity, however only at very high concentrations, while co-administration of CATH-2 and D-Arg significantly reduced this toxic effect (**Figure 6**). The data demonstrate that the interaction between D-arginine and CATH-2 actively mitigates toxicity rather than merely existing as a passive observation.

More importantly, no significant toxicity was observed with respect to udder cells exposed to milk alone or to milk containing the D-Arg/CATH-2 combination, even at the highest concentrations (**Figure 7**). These concentrations were far above the MIC in raw milk (**Figure 8**). Since this most closely resembles the *in vivo* situation, safety of potential intramammary application seems very well possible from a toxicity point of view.

The enhancing effect of D-Arginine and D-Lysine when combined with CATH-2 likely arises from synergistic mechanisms rather than direct binding. Indeed, we assessed binding between D-Arg and CATH-2 using isothermal titration calorimetry, but no detectable heat production indicative of binding could be detected (**Supplementary Figure S7**). D-Arg and D-Lys are the only two positively charged D-amino acids. The positive charge indicates that they can bind more easily to the negatively charged bacterial membrane (Wilson et al., 2001) then to the positively charged CATH-2. The compounds may facilitate entry into the bacterial cell for each other.

For most antimicrobial peptides, the mechanism of action involves bacterial membrane-permeabilization. The positively charged peptide electrostatically binds negatively charged membranes after which the peptides insert themselves in the membrane creating pores or destabilizing the membrane such that they become porous (Mookherjee et al., 2020). This mechanism applies also to CATH-2, although it has also been shown that this peptide can translocate into bacterial cells with minimal observable membrane damage at low concentrations. This enables CATH-2 to also access intracellular targets (Schneider et al., 2016). There is much less information on the antibacterial mechanism of D-amino acids, but tolerance to D-Arginine in bacteria has been associated with mutations in the phosphate transport and chaperone systems. This implies D-Arg targets cytoplasmic processes (Alvarez et al., 2018). The combination may allow D-Arg/D-Lys to exploit CATH-2-induced membrane perturbations to reach these intracellular sites without extracellular degradation. Synergistic effects could also stem from dual targeting: CATH-2 disrupts membrane integrity, while D-Arg/D-Lys interfere with metabolic pathways such as phosphate homeostasis or protein folding. This multi-pronged action might reduce the threshold for bacterial killing compared to either component alone.

It is unclear why the synergistic effect of CATH-2 and D-Arg in milk is even larger than in bacterial growth media. Milk is known to contain proteases, especially plasmin (derived from the inactive plasminogen), but in our opinion it is unlikely that the effect could be related to protease inactivation by the combination of CATH-2 and D-Arg. Plasmin is a trypsin like protease that cleaves peptide sequences indeed containing arginine or lysine but no reports of inhibition by free D-amino acids have been reported. An alternative explanation for synergy is that in milk the bacterial membrane might be covered or protected by milk components making it difficult for CATH-2 to penetrate the bacterial membrane, while the positively charged D-amino acid, due to electrostatic interactions with the negatively charged bacterial membrane can sensitize the membrane for CATH-2 penetration. However, this is all speculative and the exact mechanism of synergistic action is a subject for follow up studies.

The fast-killing kinetics and reduced quantities for killing bacteria observed for the CATH-2/D-Arg combination in raw milk reduces the hurdles of cost and instability of AMPs in physiological environments that have hampered much of the application of AMPs into useful drugs. It is anticipated that the production cost of the CATH-2/D-Arg formulation, intended for intramammary administration immediately post-milking in farm animals diagnosed with mastitis, will remain below €10 per treated animal, and if efficient large scale peptide production is achieved much lower than that.

Our research shows that a combination of natural compounds, positively charged D-amino acids and the antimicrobial peptide CATH-2, effectively eradicate mastitis-causing bacteria in raw cow milk. These novel synergistically working antibacterial compounds offer a promising potential for treating bacterial infections, particularly those caused by antibiotic-resistant strains and moves us nearer to combating the global threat of antibiotic resistance.

## Data Availability

The original contributions presented in the study are included in the article/**Supplementary Material**. Further inquiries can be directed to the corresponding author.
