# Supplementary material for: Synergistic antibacterial effect of CATH-2 and D-amino acids against mastitis causing gram-positive bacteria
Source: Front Cell Infect Microbiol. 2026 May 29;16:1819326. doi: 10.3389/fcimb.2026.1819326 (PMC13260288; doi:10.3389/fcimb.2026.1819326)
Supplement: Supplementary file 1 [file SupplementaryFile1.docx]

**Supplementary figures**


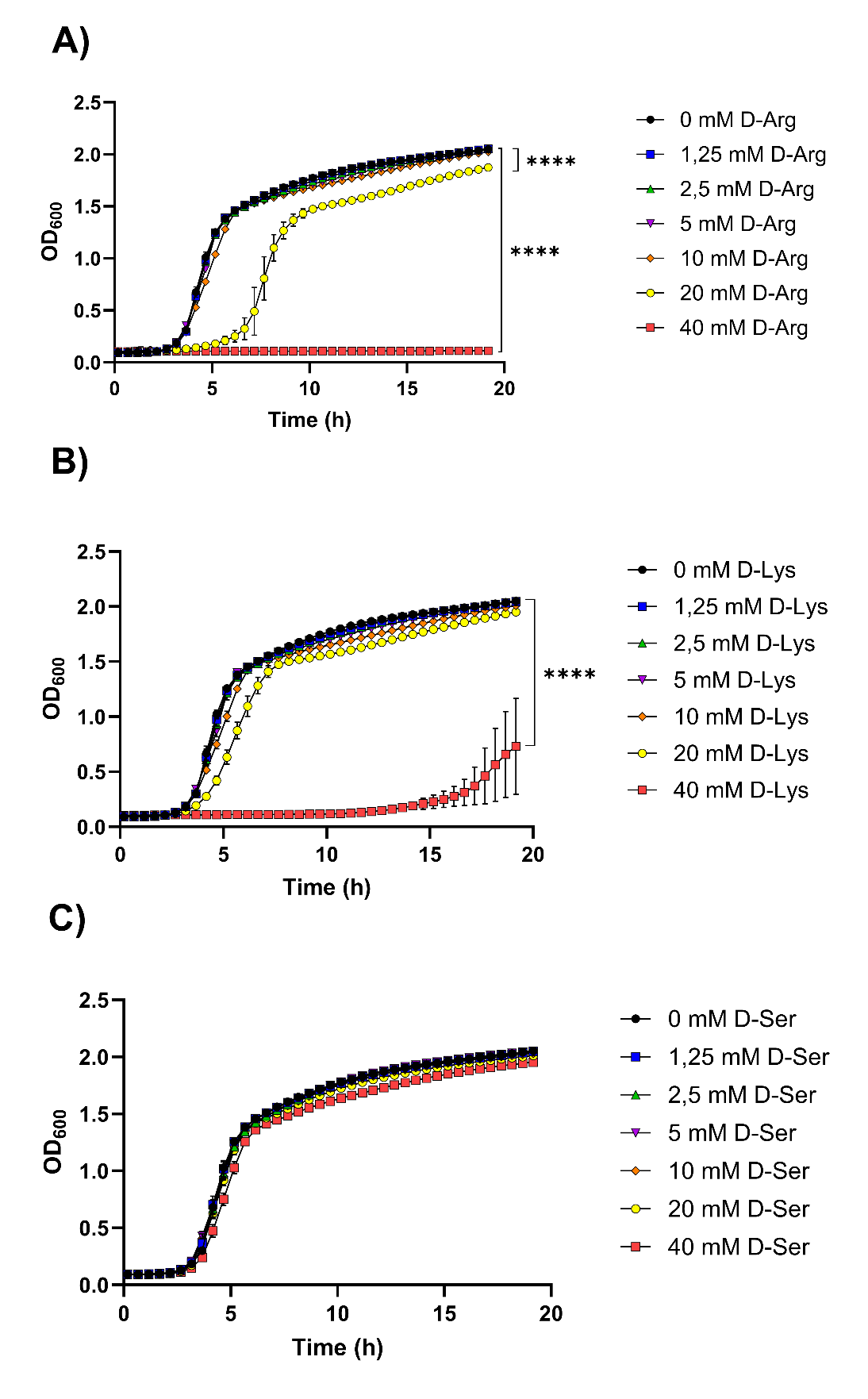


***Figure S1.*** *Effect of D-amino acids on growth of S. aureus.*

*S. aureus JHs58 was incubated with different concentrations of D-Arg, D-Lys and D-Ser at 37 °C and growth was determined by measuring OD_600_ every 10 min. Data are represented as mean ± SEM of triplicate independent experiments. P-value,* ****P < 0.0001.


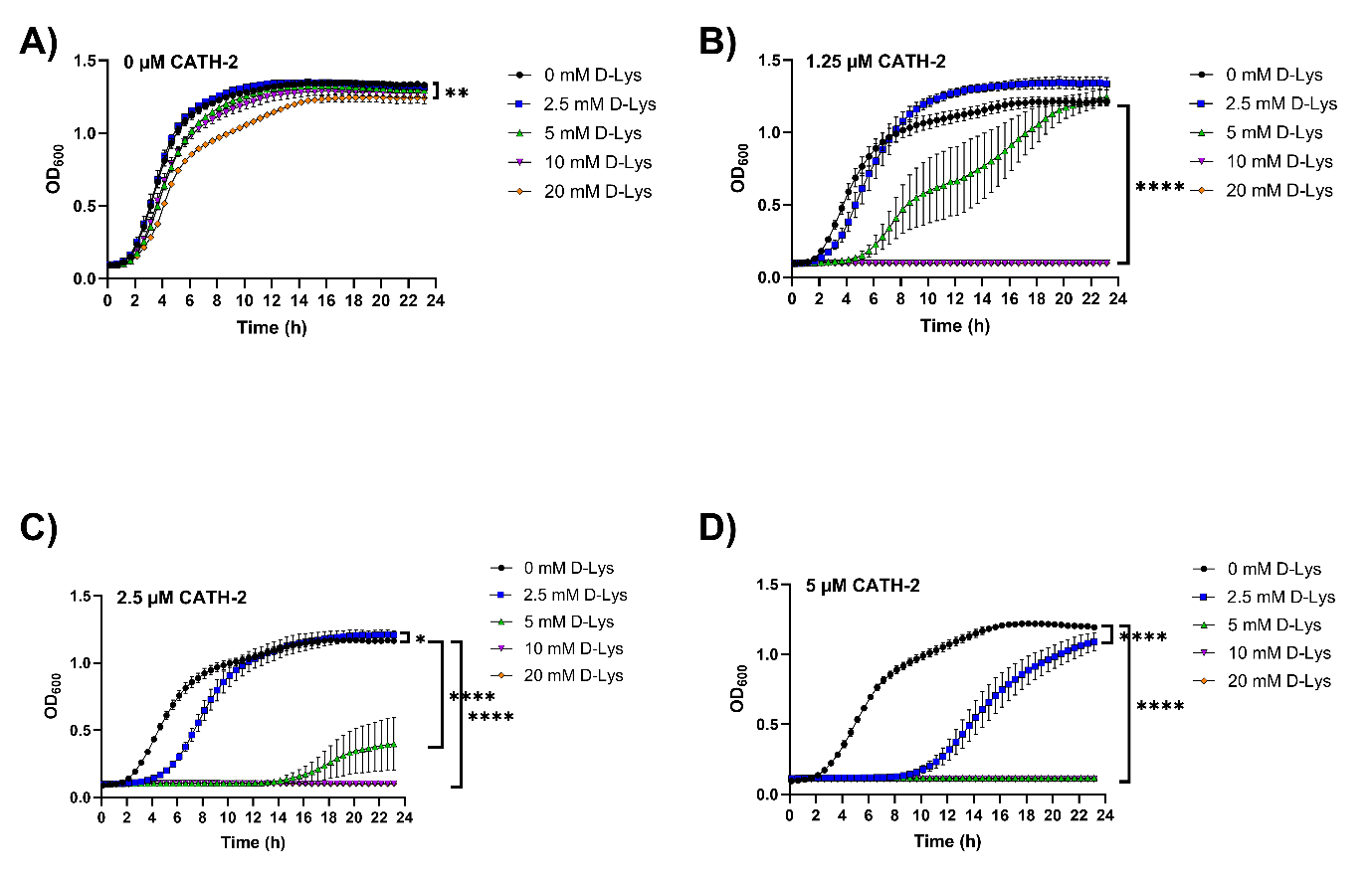


***Figure S2.*** *Antibacterial effect of D-lysine and CATH-2 (growth curves).*

*S. aureus was incubated with different concentrations of D-lys as indicated and with A)* *0* *µM CATH-2; B) 1.25 µM CATH-2; C) 2.5 µM CATH-2 and D) 5 µM CATH-2* *at 37 °C and growth was determined by measuring OD_600_ every 10 min. Data are represented as mean ± SEM of three independent experiments. P-value,* *P < 0.05, **P < 0.01, ***P < 0.001, ****P < 0.0001.


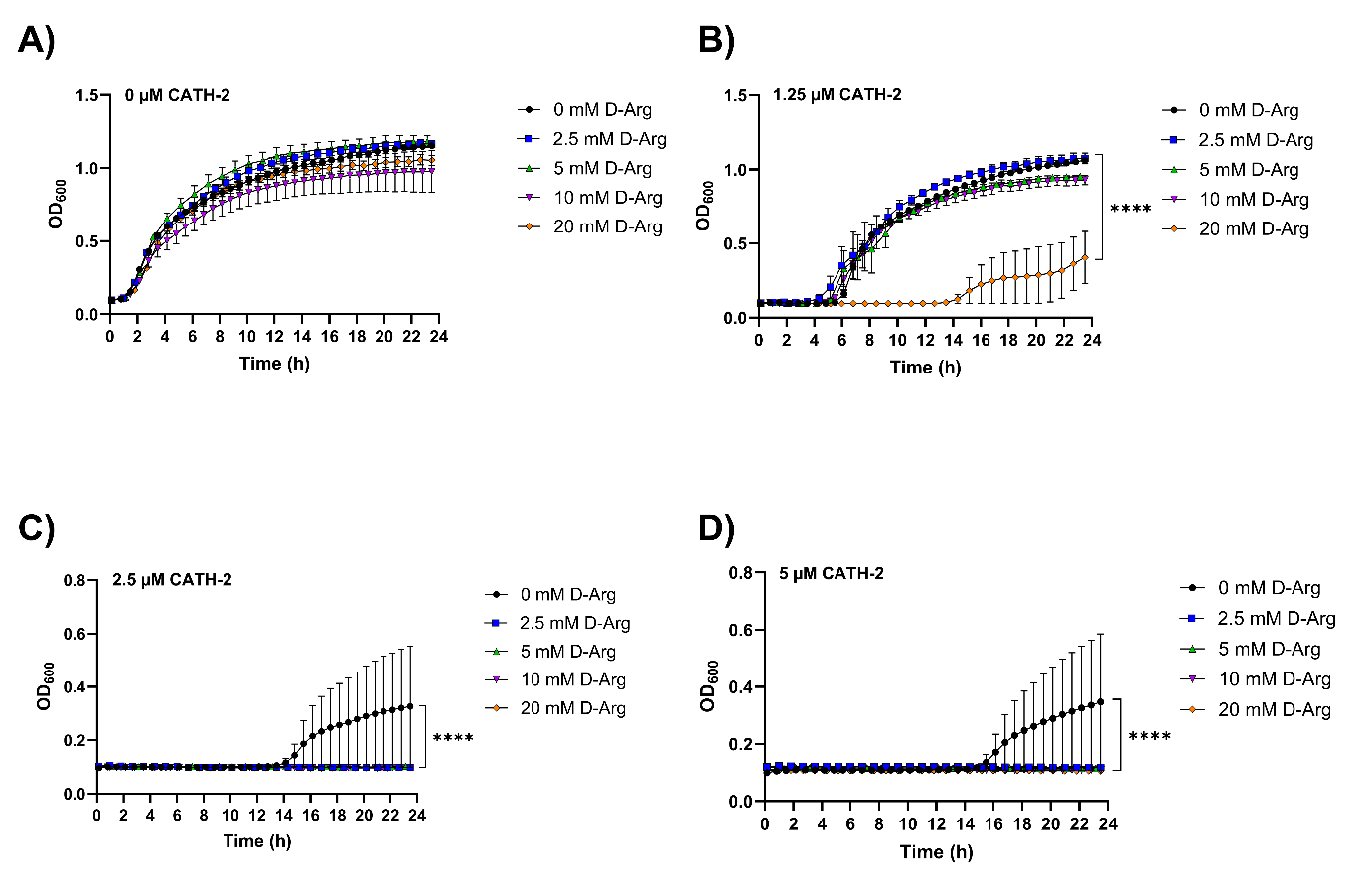


***Figure S3.*** *Antibacterial effect of D-Arginine and CATH-2 on E. coli (growth curves).* *E. coli was incubated with different concentrations of D-Arg as indicated and with A)* *0 µM CATH-2; B) 1.25 µM CATH-2; C) 2.5 µM CATH-2 and D) 5 µM CATH-2 at 37 °C and growth was determined by measuring OD_600_ every 10 min. Growth curves are represented as mean ± SEM of three independent experiments. P-value*, ****P < 0.0001.


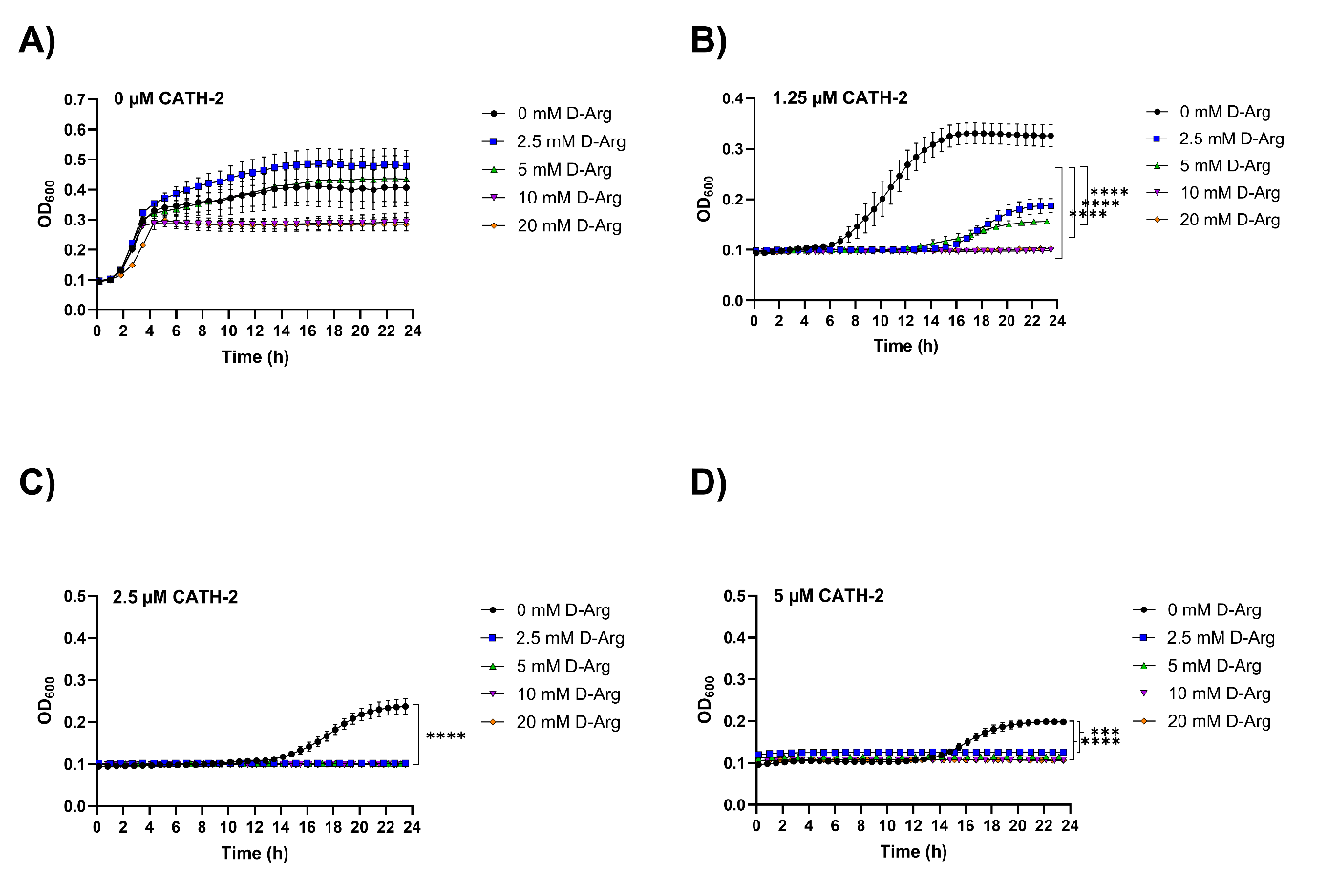


***Figure S4.*** *Antibacterial effect of CATH-2 and D-Arginine on S. uberis****.*** *S. uberis* 233 h4c1 *was incubated with different concentrations of D-Arg as indicated and with A) 0 µM CATH-2; B) 1.25 µM CATH-2; C) 2.5 µM CATH-2 and D) 5 µM CATH-2 at 37 °C and growth was determined by measuring OD_600_ every 10 min. Growth curves are represented as mean ± SEM of three independent experiments. P-value*, ***P < 0.001, ****P < 0.0001.


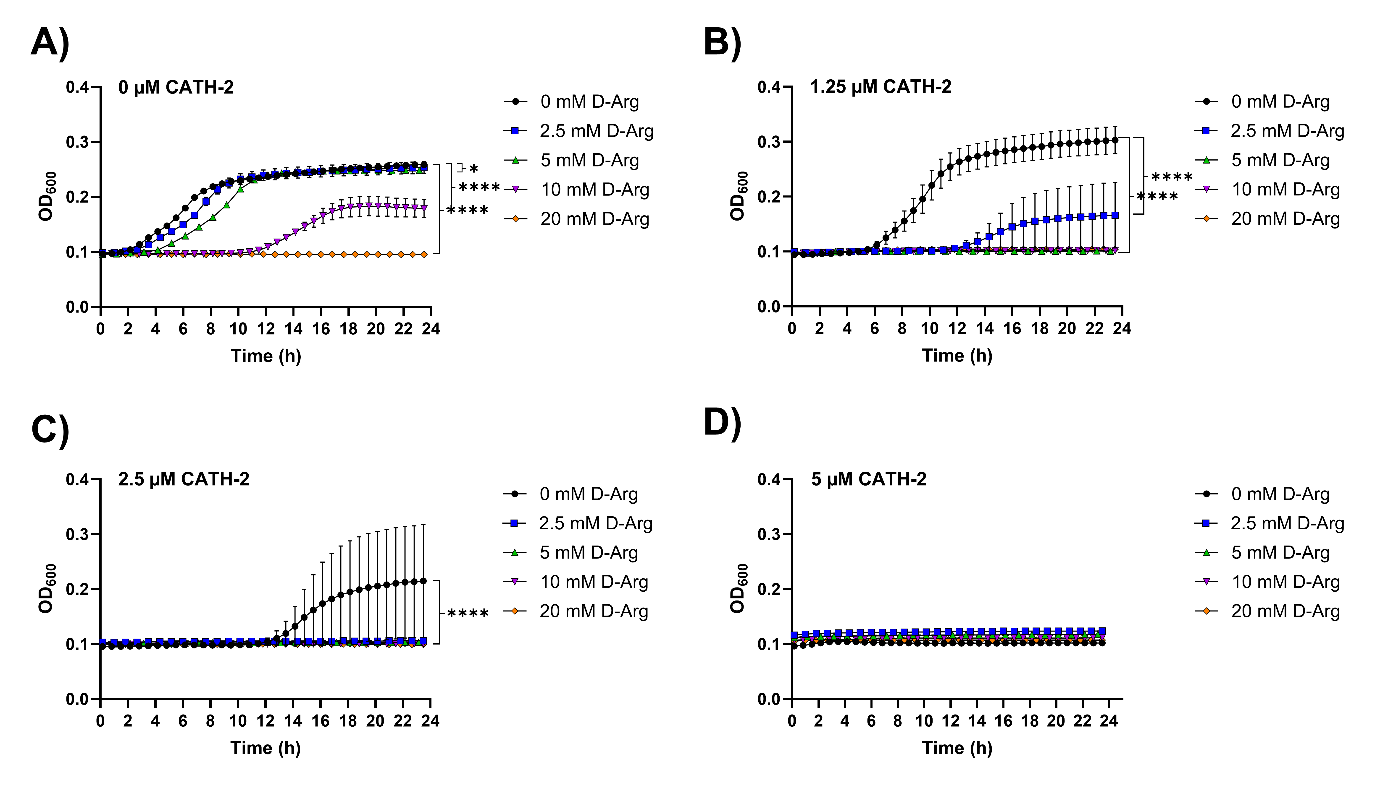


***Figure S5.*** *Antibacterial effect of CATH-2 and D-Arginine on S. agalactiae.*

*S. agalactiae was incubated with different concentrations of D-Arg as indicated and with A) 0 µM CATH-2; B) 1.25 µM CATH-2; C) 2.5 µM CATH-2 and D) 5 µM CATH-2 at 37 °C and growth was determined by measuring OD_600_ every 10 min. Growth curves are represented as mean ± SEM of three independent experiments. P-value,* *P < 0.05, ****P < 0.0001.


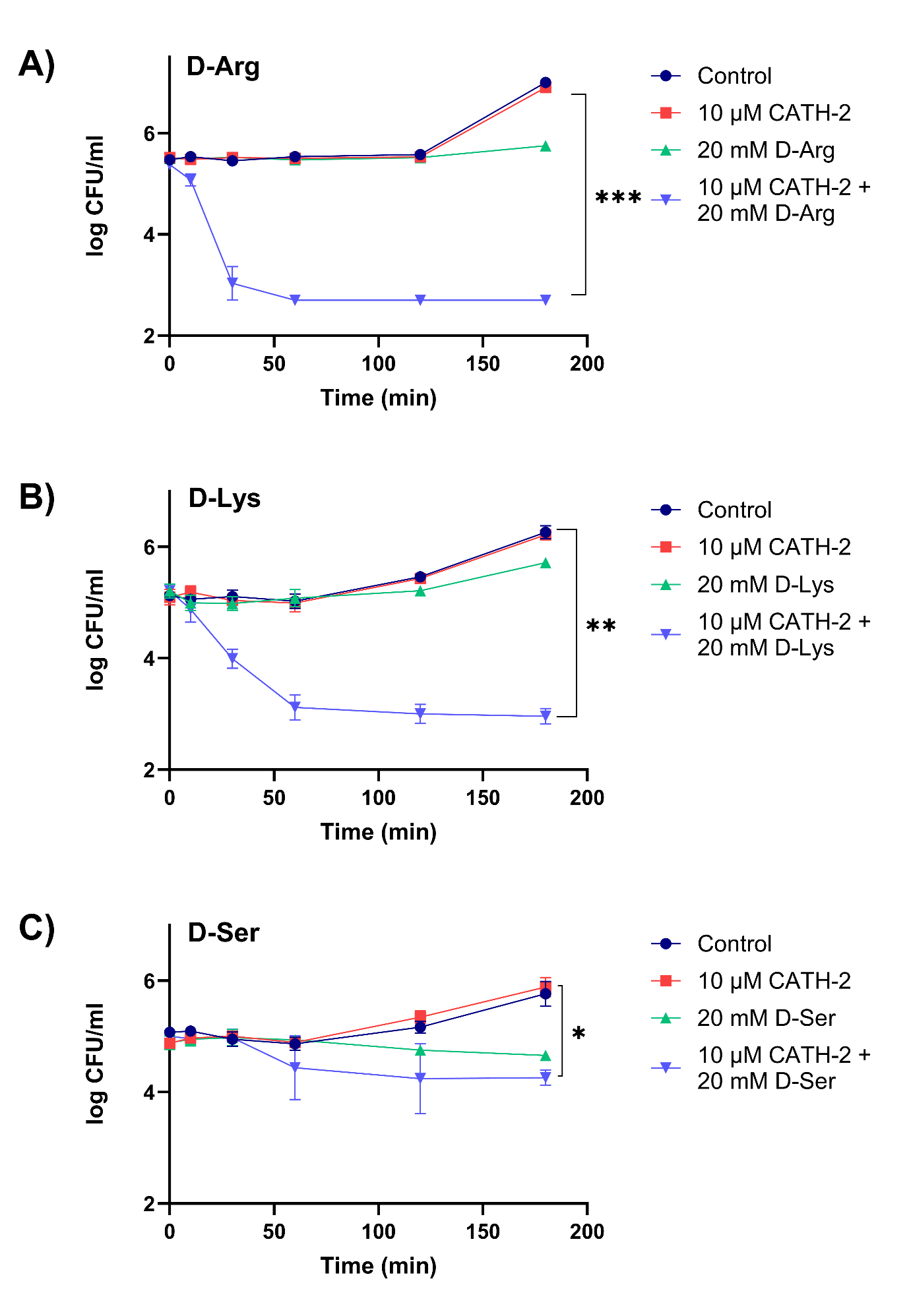


***Figure S6.*** *Kinetics of antimicrobial activity of CATH-2 with and without D-Arg, D-Lys or D-Ser on S. aureus ATCC25923 in raw milk.* *Bacteria were incubated for a duration of 3 h with CATH-2 and/or D-Arg dissolved in raw milk. During this period, multiple samples of all suspensions were taken and plated on TSA plates. Viable bacteria were counted. Data are represented as mean and SEM of triplicate independent experiments (n=3). Killing for each suspension was compared to the control suspension by one-way Anova followed by Dunnett’s multiple comparisons test. P-value.,* *P < 0.05, **P < 0.01, ***P < 0.001.


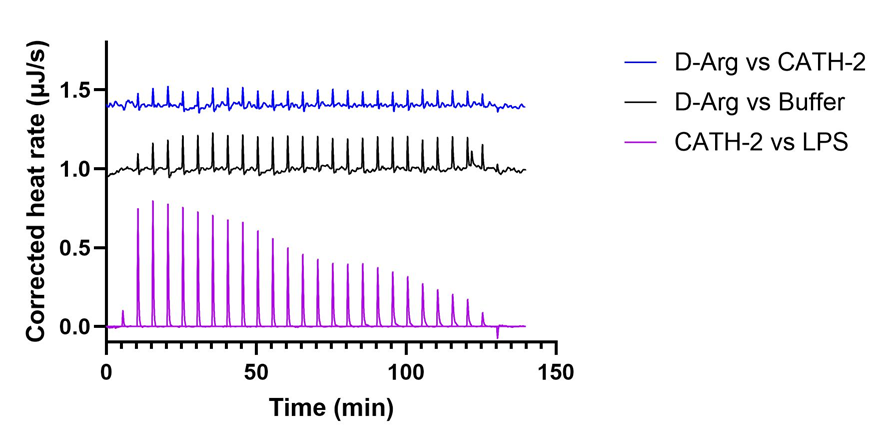


**Figure S7**: ITC spectra fail to indicate interaction between CATH-2 and D-Arg. Blue line: 1 mM D-Arg was titrated in 100 µM CATH-2 solution in PBS. Black: 1 mM D-Arg was titrated in PBS. Purple: 200 µM CATH-2was titrated in 10 µM LPS from Pseudamonas aeruginosa. Heat rates were recorded on a Low Volume NanoITC (TA Instruments-Waters LLC, 413 New Castle, DE, USA).
